# An analysis of the distribution of bone and soft tissue sarcoma diagnoses and their disparities in Southwest Germany: a multicenter approach

**DOI:** 10.3389/fonc.2025.1592004

**Published:** 2025-10-22

**Authors:** Branko Calukovic, Katrin Benzler, Mary E. Carter, Adrien Daigeler, Johannes T. Thiel, Jens Jakob, Bernd Kasper, Gerlinde Egerer, Leonidas Apostolidis, David Braig, Simone Hettmer, Claudia Blattmann, Markus Knott, Lars Zender, Christoph K. W. Deinzer

**Affiliations:** 1 Department of Internal Medicine VIII - Medical Oncology and Pneumology, Medical University Hospital Tübingen, Tübingen, Germany; 2 Center for Soft Tissue Sarcomas, GIST and Bone Tumors (ZWS) of the University Hospital Tübingen and the Comprehensive Cancer Center (CCC) Tübingen-Stuttgart, Tübingen, Germany; 3 DFG Cluster of Excellence 2180 'Image-Guided and Functional Instructed Tumor Therapy' (iFIT), University of Tübingen, Tübingen, Germany; 4 Department of Hand, Plastic, Reconstructive and Burn Surgery, BG-Unfallklinik Tübingen, Eberhard Karls University of Tübingen, Tübingen, Germany; 5 Sarcoma Unit, Mannheim University Medical Center, University of Heidelberg, Mannheim, Germany; 6 Sarcoma Center Heidelberg, Heidelberg, Germany; 7 Department of Hematology, Oncology and Rheumatology, University Hospital Heidelberg, Heidelberg, Germany; 8 Department of Medical Oncology, National Center for Tumor Diseases (NCT) Heidelberg, Heidelberg University Hospital, Heidelberg, Germany; 9 Sarcoma Center of the Comprehensive Cancer Center Freiburg (CCCF), Freiburg, Germany; 10 Department of Plastic and Hand Surgery, Medical Center - University of Freiburg, Faculty of Medicine, University of Freiburg, Freiburg, Germany; 11 Division of Pediatric Hematology and Oncology, Department of Pediatric and Adolescent Medicine, University Medical Center Freiburg, University of Freiburg, Freiburg, Germany; 12 Sarcoma Center Stuttgart Cancer Center (SCC), Klinikum Stuttgart, Stuttgart, Germany; 13 Department of Paediatric Hematology and Oncology, Olgahospital, Stuttgart Cancer Center (SCC), Klinikum Stuttgart, Stuttgart, Germany; 14 Stuttgart Cancer Center - Tumorzentrum Eva Mayr-Stihl, Klinikum Stuttgart, Stuttgart, Germany; 15 Department of Hematology and Oncology, Klinikum Stuttgart, Stuttgart, Germany

**Keywords:** soft tissue sarcoma, bone sarcoma, white-spot analysis, cancer registry, geographic disparities, specialized sarcoma center, real world data

## Abstract

**Introduction:**

Sarcoma is a rare and highly heterogeneous family of mesenchymal tumors. The experience and interdisciplinary approach of specialized high-volume sarcoma centers has a significant impact on disease treatment and outcome for patients. The aim of this retrospective, real-world, multicenter study was to evaluate geographic distribution of sarcoma cases in Southwest Germany and visually depict possible underrepresented areas of sarcoma primary diagnoses. Such descriptive information may indirectly guide future referral patterns and outreach activities of specialized sarcoma centers.

**Methods:**

The absolute number and incidence of sarcoma patients obtained from the Baden-Württemberg Cancer Registry were compared with the data from five individual, high-volume, specialized sarcoma centers. Furthermore, we used a “White-Spot Analysis” as a novel cost-effective approach in epidemiological and public health research for analyzing health care coverage in sarcoma care.

**Results:**

A total of 4,087 sarcoma patients living in the German Federal State of Baden-Württemberg between 2019 and 2022 were included in this study. Of these, 1,650 patients (40%) were treated primarily in specialized sarcoma centers whilst 2,437 patients (60%) received treatment for sarcoma outside of the five main high-volume centers, in underrepresented areas identified through White-Spot Analysis. The sarcoma incidence in Baden-Württemberg was calculated with our data to be 9.18/100,000 inhabitants per year.

**Discussion:**

In future, the access to high-volume centers needs to be facilitated in order to minimize the observed discrepancies between treatment in specialized sarcoma centers and low-volume centers in Southwest Germany. Our analysis highlights such discrepancies and may support future efforts to improve outcomes for sarcoma patients.

## Graphical Abstract

**Figure:**
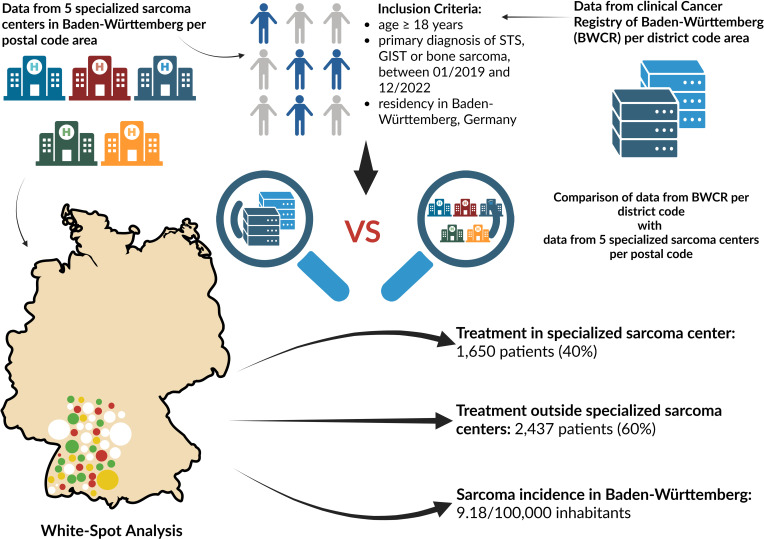
The graphical abstract depicts the inclusion criteria and provides a comparative overview of the two data sources of the analysis: aggregated data from five specialized sarcoma centers in Baden-Württemberg integrated with the White-Spot Analysis, and data derived from the clinical Cancer Registry of Baden-Württemberg (BWCR). The results are summarized in the lower right corner. Reference: Created in BioRender. Lauer, U. (2025) https://BioRender.com/s5kwo76.

## 1 Introduction

Sarcomas are defined as a group of rare and heterogeneous malignancies of mesenchymal origin, though individual subtypes arise from diverse mesenchymal lineages and some from uncertain histogenesis (1, 2). They encompass multiple, unique histological subtypes, can occur at any age and location in the human body and exhibit unique clinical behaviors distinct from epithelial malignancies (3). Sarcomas are typically divided into two major groups: bone sarcoma and soft tissue sarcoma. On the basis of histological, clinical and molecular-genetic characteristics further subtypes can be differentiated (4). Due to improved understanding of morphologic, immunohistochemical and molecular-genetic characteristics, the World Health Organization (WHO) updated its Classification of soft tissue tumors (5^th^ edition) in 2020, to enable standardization of the diagnostic process (5).

Epidemiologically, the majority of sarcoma cases occurs sporadically, though a small number of cases tend to arise as a part of familial cancer syndromes, such as Li-Fraumeni or Werner Syndrome (6). Sarcomas constitute about 15% of all pediatric and less than 1% of all solid malignancies in the adult population world-wide (6). Unlike bone sarcomas, which exhibit rather stable incidence rates across all age groups with a noticeable increase of rates in adolescents and young adults due to osteosarcoma and Ewing’s sarcoma, soft-tissue sarcomas (STS) typically show a biphasic incidence curve with marked increases in children under 5 years of age and adults over 70 years (2). According to the German National Cancer Register data, a total of 4,610 new STS (2,190 women and 2,420 men) and 570 new bone sarcoma (360 women and 510 men) cases were detected in 2020 (7), with incidence steadily rising over the recent decades (7). In the year 2020, national cause-of-death statistics report STS as being responsible for 1,835 (908 women and 927 men) and bone sarcoma for 440 (183 women and 257 men) deaths in Germany (7).

Prognosis varies widely across sarcoma subtypes, ranging from relatively favorable outcomes in selected GIST cases treated with targeted therapies to poorer survival in metastatic osteosarcoma, as reported in the EURAMOS-1 cohort (8). The most important prognostic variables for sarcoma are tumor grading, tumor staging and tumor location (9, 10). When a sarcoma is suspected, imaging diagnostics of local spread should be performed before biopsy in accordance with guidelines (9–11). Since an adequate biopsy is a key step in diagnosing and classifying sarcoma, such interventions should be conducted in sarcoma reference centers and/or within reference networks sharing multidisciplinary expertise and treating a relevant number of sarcoma patients annually (“high-volume centers”) (9, 10, 12). In most studies case volume is used as an indicator of expertise. The definition of high-volume centers can differ from study to study. Some studies consider those in the top 1% for case volume whereas others generally include centers treating between 5 and 20 new surgical cases annually (13).

Sarcoma centers according to the German Cancer Society are required to undergo a certification process and meet defined quality criteria, such as a multidisciplinary approach (e.g. weekly interdisciplinary tumor board meetings), the volume of patients and appropriate facilities for proper application of clinical practice guidelines, recording and publication of outcomes (9, 10, 12). The certification process and definition of the quality criteria in Germany is managed by the German Cancer Society. Furthermore, such centers are usually involved in ongoing clinical trials, which may lead to the enrolment of patients and an offer of potentially novel therapeutic options. Several studies, such as NETSARC study which included 3,227 sarcoma patients in France, or a nationwide study from the Netherlands, or studies from the USA and the United Kingdom, report significantly better outcomes for sarcoma patients when managed in sarcoma reference centers involving multidisciplinary medical teams (8, 14–18). According to ESMO guidelines, the referral of sarcoma patients to appropriate specialized centers cannot be emphasized enough (9, 10).

After histological confirmation with tumor grading, sarcoma diagnostics should be completed with imaging of a systemic spread (9, 10). Sarcoma treatment is complex due to the cancer’s heterogeneity and should be discussed and decided in an interdisciplinary setting, such as certified or specialized sarcoma reference center (11). In most cases, treatment requires a multimodal approach and consists of surgery, radio- and chemotherapy, which could be applied in a neoadjuvant, adjuvant and/or palliative setting. In addition, in some cases these treatment options can be supplemented by loco-regional deep hyperthermia for some cases of STS (19).

In light of this complexity and a strong recommendation for treatment in a specialized center, we aimed to perform a retrospective study analyzing the geographic distribution of sarcoma cases in Southwest Germany. We first generated a topographic depiction of patients diagnosed with sarcoma based on district codes (in German “Amtlicher Gemeindeschlüssel”) from the federal Cancer Registry of Baden-Württemberg for the years 2019 to 2022 and data obtained from the five main high-volume specialized sarcoma centers in Southwest Germany (Mannheim, Heidelberg, Freiburg, Stuttgart and Tübingen). We then compared the topographical distributions of sarcoma patients to identify geographically “white spots” or areas that are possibly underrepresented in sarcoma care. Our ultimate goal with this study is to improve the quality of sarcoma treatment in Southwest Germany by depicting such possible white spots in sarcoma care, which could lead to optimization of the outreach activities of the five specialized sarcoma centers and which could increase the number of sarcoma patients treated in specialized high-volume centers with expertise in sarcoma care and therefore improve the medical outcomes for those patients.

## 2 Materials and methods

### 2.1 Inclusion and exclusion criteria

Patients who met the following inclusion criteria were eligible for our study:

- age ≥ 18 years.
- primary diagnosis of soft tissue sarcoma (STS), gastrointestinal stromal tumor (GIST) or bone sarcoma (according to histology codes according to WHO; (see **Supplementary Table 1**) between January 2019 and December 2022.
- resident in the German Federal State of Baden-Württemberg.

The data from these patients were used to depict the number of sarcoma patients with diagnosis in Southwest Germany.

The study was conducted using retrospective and anonymized data according to the declaration of Helsinki and complied with both the local General Data Protection Regulation (GDPR) and the Federal State Data Protection Laws (“Landesdatenschutzgesetz Baden-Württemberg”). The study protocol was approved by the Ethics Committee of the Eberhard Karls University and the University Hospital Tübingen (No. 093/2024BO2).

### 2.2 Collection of data from clinical cancer registry of Baden-Württemberg and from five specialized sarcoma centers

We obtained the numerical anonymized data of all sarcoma primary diagnoses from the Baden-Württemberg Cancer Registry (BWCR) according to district codes for the four-year time span from January 2019 to December 2022. The data were screened for patients that met the defined inclusion criteria of our study. We primarily analyzed the absolute number and incidence of patients with sarcoma primary diagnosis for district codes during the observation period. Additional factors such as the number of inhabitants and size of the districts were considered and factored in during analysis of the data (see **Supplementary Table 2**).

Population data for community codes of Baden-Württemberg as of 31 December 2022 were obtained from an online database of Statista Research Department GmbH, Hamburg, Germany (20).

The numbers of sarcoma primary diagnoses for each postal code area were retrieved anonymously from the specialized sarcoma centers in Mannheim, Heidelberg, Freiburg, Stuttgart and Tübingen. At the time of data collection, the two sarcoma centers in Mannheim and Tübingen were certified by the German Cancer Society. Key factors for certification, in addition to primary case numbers, include metrics such as the surgical expertise of individual surgeons for STS and bone sarcomas, pre- and post-therapeutic presentations in the interdisciplinary tumor board, R0 resection rates, the proportion of study patients, and psycho-oncological screenings (21). The other participating sarcoma centers, such as Heidelberg, Freiburg, and Stuttgart, had already specialized in sarcoma treatment in the past as maximum care providers or university hospitals in Baden-Württemberg. Therefore, based on case numbers, we assume comparable and adequate sarcoma care, even though this was not conclusively verified for all the aforementioned centers at the time of data collection. The Heidelberg and Stuttgart Sarcoma Center have since achieved certification, while the sarcoma center in Freiburg which do not has other maximum care providers in the catchment area, is currently still undergoing the certification process by the German Cancer Society. The numbers of inhabitants of each postal code area were downloaded from an open-source database which included German population data from German statistical offices of the initiative “Zensus 2011” and the German postal code areas provided by OpenStreetMap (22).

### 2.3 Analysis and visualization of results

In addition to the absolute number of sarcoma patients per district, we computed crude annual incidence rates, defined as the number of newly diagnosed patients during 2019–2022 divided by the average population during the same period, divided by 4, and expressed per 100,000 inhabitants per year. Furthermore, mean and median number of sarcoma patients of each sarcoma center with calculation of the interquartile range were determined to evaluate the regional distribution of both absolute patient numbers and incidence per postal code area. Subsequently, the data were visualized on a map of the German Federal State of Baden-Württemberg. Analysis of data was performed via Microsoft Word and Excel of Microsoft Office Professional Plus 2019 (Microsoft Corporation, Redmond, WA, USA). For visualization and illustration of the absolute number and raw incidence of sarcoma patients, QGIS, an open-source geographic information system (QGIS Development Team; under license of GNU General Public License, Version 3.32.3) was used. Citations were managed through Clarivate EndNote version 21 (Clarivate, 70 St. Mary Axe, London EC3A 8BE, United Kingdom).

## 3 Results

### 3.1 Analysis of sarcoma primary diagnoses per district code area (data from BWCR)

**Figure 1:**
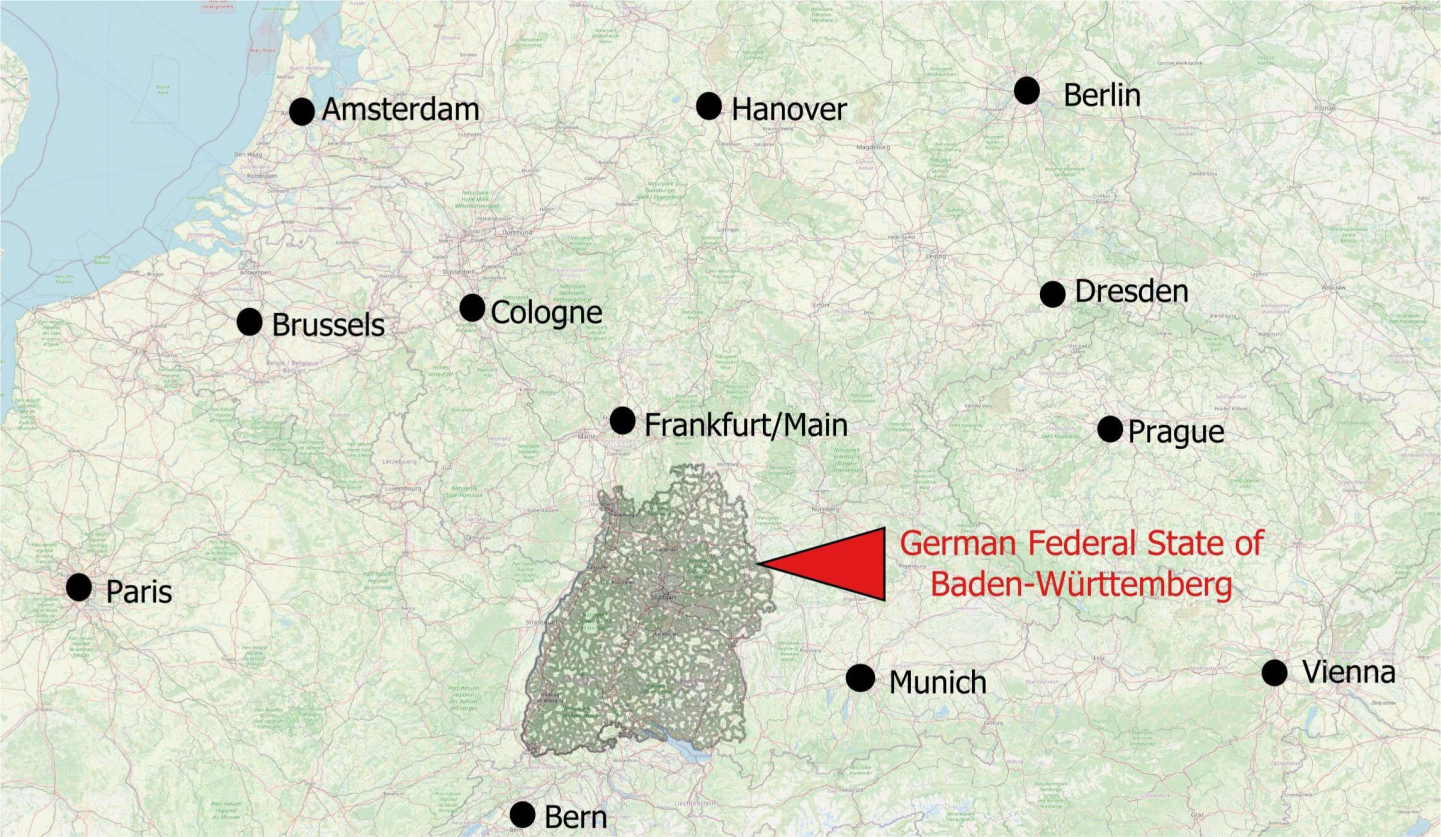
Location of the German Federal State of Baden-Württemberg in Europe. The map illustrates the geographic setting of the study area in a European context.

Baden-Württemberg is a German Federal State and is divided into 44 districts (35 rural districts and 9 independent cities) and 1,194 postal code areas, covering a total area of 35,747 km^2^ and a population of 11,280,257 inhabitants. The location of Baden-Württemberg in Europe is shown in **Figure 1**. A total of 4,087 patients with a first diagnosis of sarcoma, consisting of 269 bone tumors and 3,818 soft tissue sarcomas, were observed in this region during the study period.

**Figure 2:**
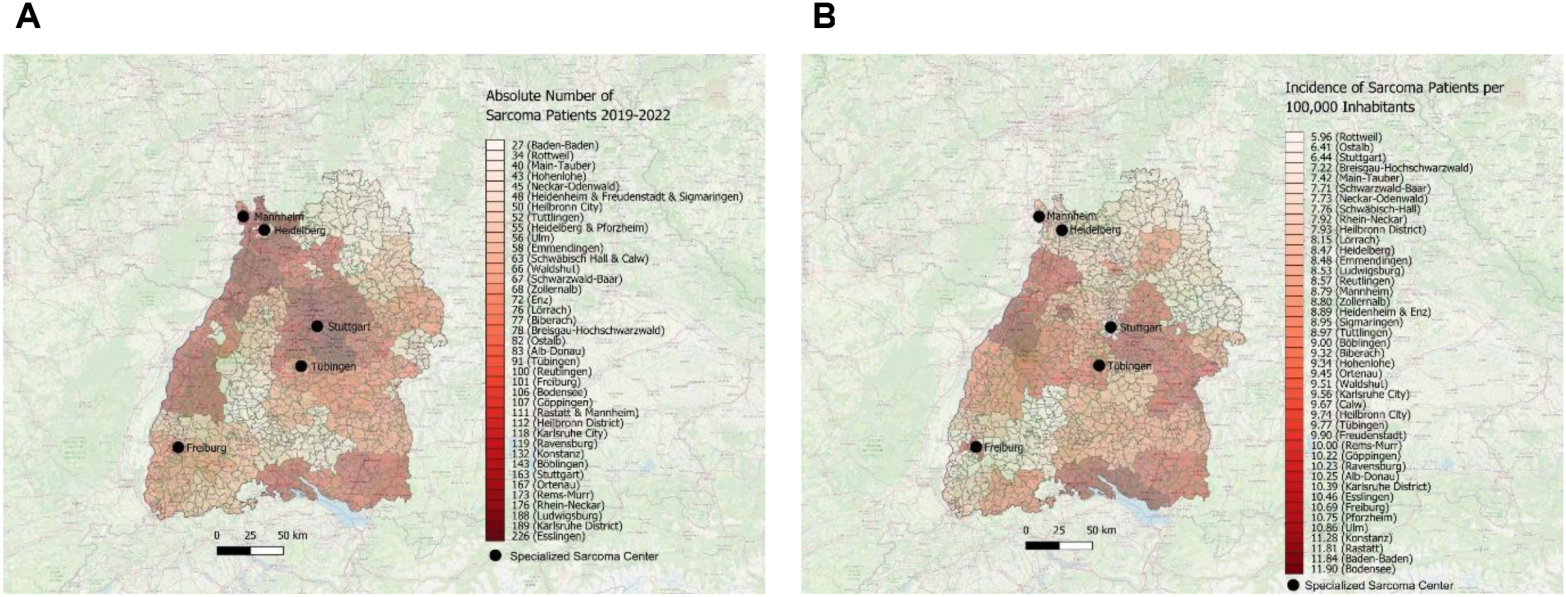
**(A)** Visual depiction of distribution of sarcoma first diagnoses with absolute number of patients across the districts in Southwest Germany for the time period 2019–2022 using the map of Baden-Württemberg and red scale. **(B)** Visual depiction of incidence with distribution of sarcoma first diagnoses per 100,000 inhabitants across the districts in Southwest Germany for the time period 2019–2022 using the map of Baden-Württemberg and red scale.

The absolute number of patients during the observation time were color-coded on a red scale to illustrate the distribution of absolute number of sarcoma patients in Baden-Württemberg. As depicted in **Figure 2A**, the district of Esslingen (district code 08116) exhibited the highest absolute number of sarcoma patients, followed by the dark red color-coded municipalities of Central and North West Baden-Württemberg, such as Ludwigsburg (district code 08118), Karlsruhe (district code 08215) and Rhein-Neckar (district code 08226). Importantly, one can observe white and light-red color-coded regions in the northeastern and midwestern parts of the Federal State of Baden-Württemberg. The lowest absolute number of patients was detected in the districts of Baden-Baden (district code 08211), followed by Rottweil (district code 08325) and Main-Tauber (district code 08128).

Next, we calculated the incidence of newly diagnosed sarcoma patients (per 100,000 inhabitants) in Southwest Germany between 2019 and 2022 and visualized the data by using a red color-coding. As depicted in **Figure 2B**, the highest incidence of newly diagnosed sarcoma patients was found in southern and western municipalities in Baden-Württemberg, such as in the district of Bodensee (district code 08435), Baden-Baden (district code 08211) and Rastatt (district code 08216). In contrast, there are vast areas in northeastern and southwestern parts of the Federal State marked with white and light red tones, such as the district of Rottweil (district code 08325) or Ostalb or the city of Stuttgart (district code 08136).

### 3.2 White-spot analysis of sarcoma primary diagnoses per postal code area (data from 5 specialized sarcoma centers)

We then obtained anonymized data regarding sarcoma primary cases from the following main specialized sarcoma centers in Southwest Germany: University Hospital Freiburg, University Hospital Heidelberg, University Hospital Mannheim, Municipal Hospital Stuttgart and University Hospital Tübingen. Only patients permanently resident in Baden-Württemberg were eligible for our multicenter study. Our study patients, treated in the 5 participating specialized sarcoma centers, came from 612 different postal code areas. The complete analyzed data points are depicted in Table 3 (see **Supplementary Materials**).

The data analysis showed a total of 1,650 eligible sarcoma patients with primary diagnosis, who have been treated in one of the participating sarcoma centers. During the observation time period, most of the sarcoma patients living in Baden-Württemberg were treated at the University Hospital Tübingen (527 patients) followed by Heidelberg (415 patients), Freiburg (301 patients), Stuttgart (253 patients) and Mannheim (154 patients) when looking at the absolute number of patients. Due to their geographical location in the northern part of Baden-Württemberg near the border to Rhineland Palatinate and Hessen, the sarcoma centers Heidelberg and Mannheim additionally treat many patients from outside Baden-Württemberg which were not part in this analysis. The University Hospital Freiburg had the highest average number of patients per postal code population per 100,000 inhabitants with 30.5 patients. In addition, the highest median number of patients with sarcoma first diagnosis per postal code population per 100,000 inhabitants with 23.9 patients could be seen at the University Hospital Freiburg.

A final postal-code specific analysis was performed using data obtained from aforementioned sarcoma centers. The analysis included absolute numbers as well as the incidence of newly diagnosed sarcoma patients in Southwest Germany. The implementation of a color on the map of Baden-Württemberg aims at depicting white spots. These white spots are characterized by a low incidence of sarcoma patients treated in one of the main five specialized sarcoma centers in Southwest Germany. To our knowledge, we are the first to create this novel “White-Spot Analysis” for sarcoma patients. A similar analysis of catchment area was only performed and published by the German WERA Consortium in 2022 for personalized cancer care and precision oncology independent of a specific cancer entity (23).

**Figure 3:**
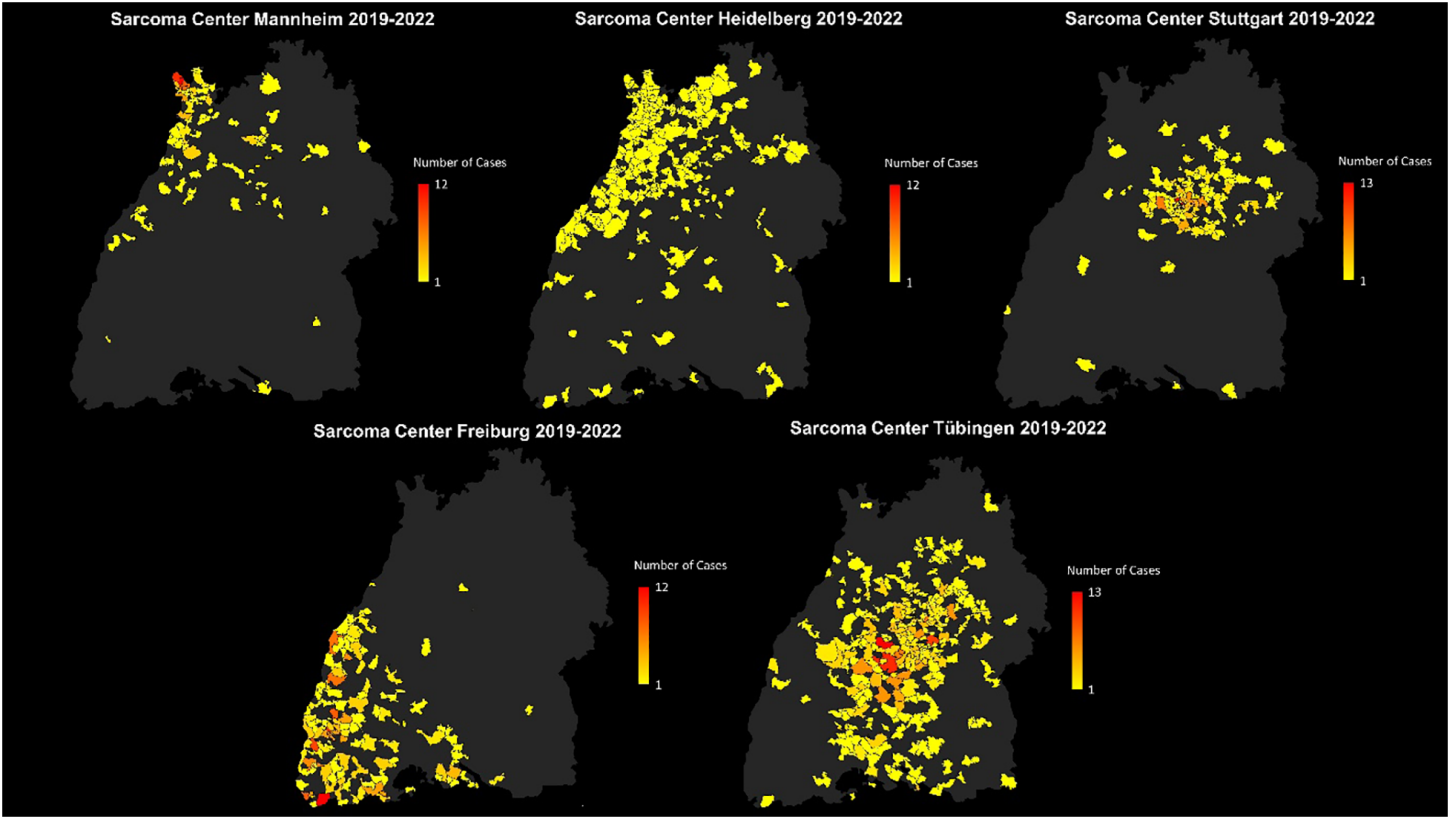
Distribution of patients with sarcoma first diagnoses treated in the sarcoma centers Freiburg, Heidelberg, Tübingen, Mannheim and Stuttgart with absolute patient numbers across the postal code areas. Each center is displayed individually to visualize its specific catchment area.

The absolute number of newly diagnosed sarcoma patients in Southwest Germany treated in one of the above-mentioned sarcoma centers was depicted using color coding based on data obtained from the specialized sarcoma centers included in the study. In **Figure 3**, each sarcoma center has been illustrated individually.

**Figure 4:**
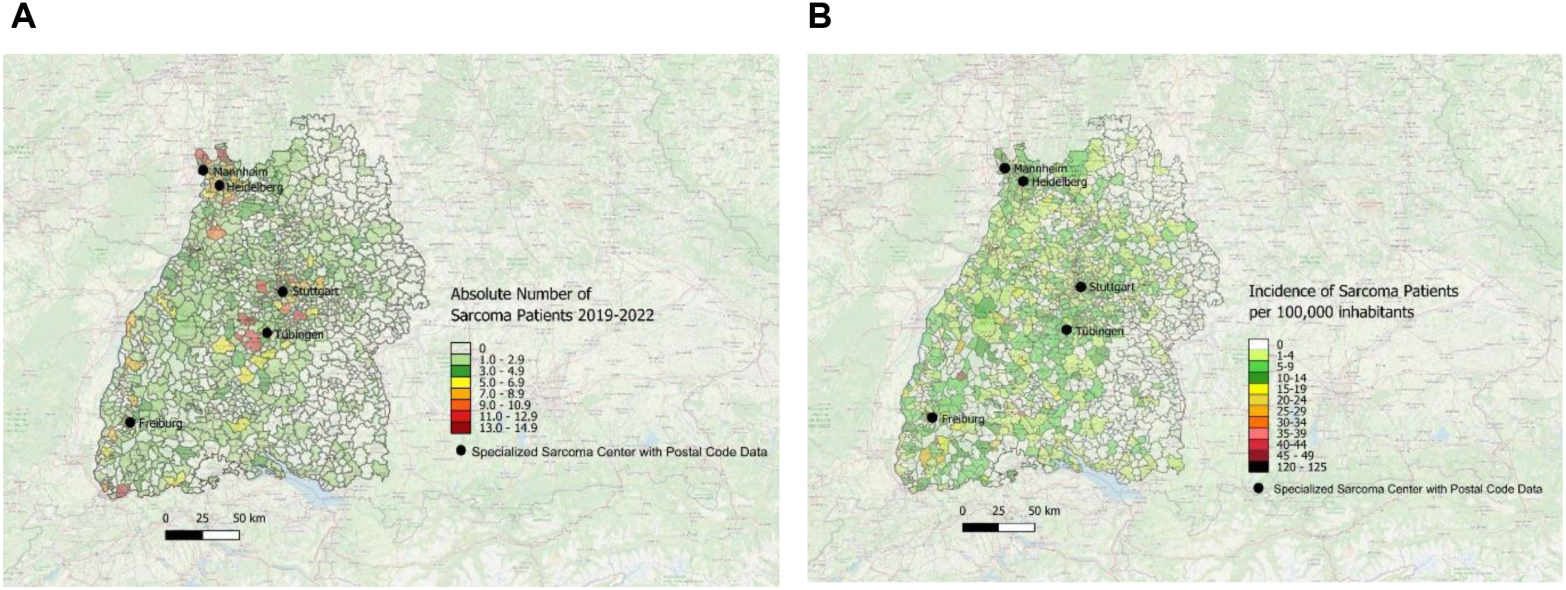
**(A)** Distribution of patients with first diagnosis of sarcoma treated in one of the five sarcoma centers with absolute patient numbers across the postal code areas in Southwest Germany for the time period 2019–2022 using the map of Baden-Württemberg and color-coding; data obtained from 5 specialized sarcoma centers. **(B)** Distribution of incidence per year patients first diagnosed with sarcoma and treated in one of the sarcoma centers across the postal code areas in Southwest Germany for the time period 2019–2022 using the map of Baden-Württemberg and color-coding; data obtained from 5 depicted sarcoma centers.

In **Figure 4A**, the data from all five centers have been merged into a single unified graphical representation. On this map, the dark red spots in central, northwestern and southwestern Baden-Württemberg, coincide with a high absolute number of sarcoma patients treated in a specialized sarcoma center. In contrast, there are multiple white and light green areas in eastern and throughout western Baden-Württemberg with a very low incidence of newly diagnosed sarcoma patients that are treated in one of the included sarcoma centers.

The incidence per year of patients first diagnosed with sarcoma (per 100,000 inhabitants) was obtained from all sarcoma centers mentioned above and is depicted in **Figure 4B**.

**Figure 5:**
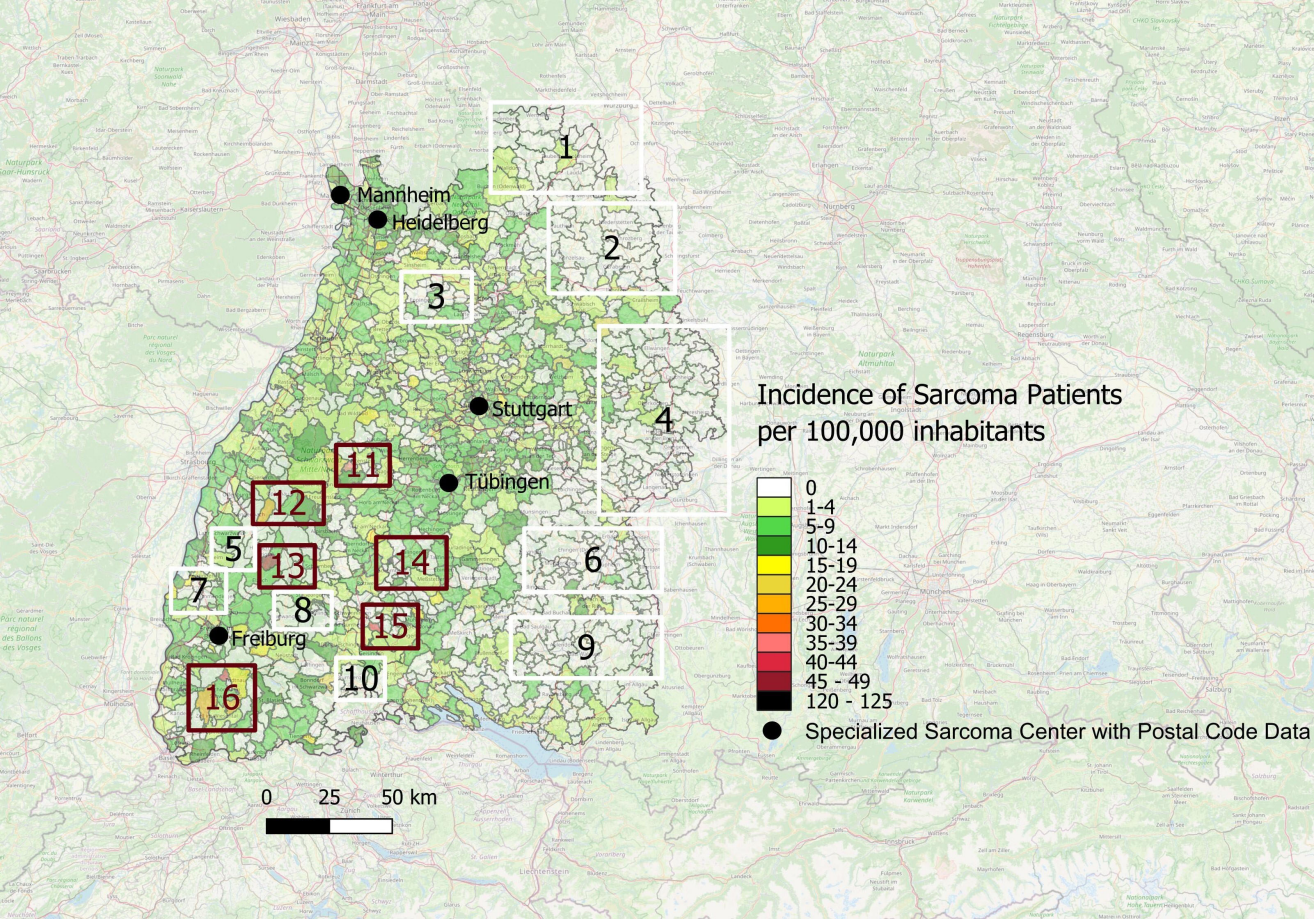
White spots and red spots representing disparities of distribution of sarcoma patients treated in one of the specialized sarcoma centers with incidence per year across the postal code areas in Southwest Germany for the time period 2019-2022; Numbering: White Spots (1-10) and Red Spots (11-16).

Furthermore, we analyzed the data depicted in the map of **Figure 4B** for the areas with the highest and lowest incidences of sarcoma diagnoses. We operationalized ‘white spots’ as postal code areas with very low or zero incidence of patients treated in one of the five sarcoma centers, indicating underrepresentation of sarcoma patients treated in specialized sarcoma centers. Accordingly, areas highlighted as white spots denote gaps in center-treated cases and not the absence of sarcoma diagnoses. As shown in **Figure 5**, there are several white spot areas in eastern Baden-Württemberg marked with white squares and numbered 1, 2, 4, 6 and 9 with a low relative number of sarcoma patients who are treated in a specialized center. Furthermore, we observed individual white spot areas throughout midwestern and southwestern Baden-Württemberg also marked with white squares and numbered 3, 5, 7, 8 and 10, where a comparatively low number of newly diagnosed sarcoma patients are treated in one of the five specialized sarcoma centers, such as the districts of Eppingen, Schwaigern, Kirchardt and Ittlingen in Heilbronn county (number 3 in **Figure 5**), the districts of Kippenheim, Seelbach, Biberach, Steinlach und Schuttertal in Ortenau county (number 5 in **Figure 5**), the districts of Endingen, Riegel and Bahlingen am Kaiserstuhl, Malterdingen and Teningen in Emmendingen county (number 7 in **Figure 5**), and the municipalities Furtwangen im Schwarzwald and Vöhrenbach in Schwarzwald-Baar county (number 8 in **Figure 5**) as well as the municipalities Stühlingen, Blumberg and Tengen in Schwarzwald-Baar and Waldshut regions (number 10 in **Figure 5**). In contrast, we observed dark red spots in central and southwestern Baden-Württemberg which illustrates areas with high incidence of patients with newly diagnosed sarcomas treated in one of the five sarcoma centers, marked with dark red squares and numbered 11-16, which are the districts of Grömbach (number 11 in **Figure 5**), Nordrach (number 12 in **Figure 5**), Mühlenbach/Schwarzwald (number 13 in **Figure 5**), Dautmergen (number 14 in **Figure 5**), Talheim/Tuttlingen (number 15 in **Figure 5**), Wieden/Schwarzwald (number 16 in **Figure 5**), Häg-Ehrsberg (number 16 in **Figure 5**) and Kleines Wiesental (number 16 in **Figure 5**).

### 3.3 Comparison of district sarcoma data from BWCR with postal code data from 5 sarcoma centers

According to the German National Cancer Register, a total of 5,480 new STS and bone sarcoma cases were diagnosed in Germany (underlying population of Germany: 83,200,000 inhabitants (24)) in 2020 (7), which represents a raw sarcoma incidence of 6.59/100,000 inhabitants for the complete area of Germany. Our analysis includes a total of 4,087 sarcoma primary cases (see **Supplementary Table 2**) over a four-year period obtained from the Baden-Württemberg Cancer Registry (underlying population of Baden-Württemberg: 11,280,000 inhabitants). Consequently, the raw sarcoma incidence in Baden-Württemberg can be calculated with our data to 9.18/100,000 inhabitants per year with pediatric sarcoma patients (age < 18 years) excluded because, in Germany, they are routinely treated in dedicated pediatric oncology centers with distinct referral pathways. Including them would not reflect adult sarcoma care structures. We observed a high disparity in the number of patients treated in all of the participating specialized sarcoma centers (1650; 40%) and compared the number of patients registered in the Baden-Württemberg Cancer Registry treated elsewhere (2,437 (60%). On the one hand, nearly all sarcoma patients living in Heidelberg and most of the patients from districts of Stuttgart, Mannheim and Tübingen were treated in a specialized sarcoma center. On the other hand, in many districts such as for example Karlsruhe, Esslingen, Göppingen, Calw, Hohenlohe and Rastatt the majority of sarcoma patients received medical treatment other than in the five specialized sarcoma centers. Disparities in sarcoma care between data of BWCR and 5 specialized sarcoma centers are depicted in Table 2 (see **Supplementary Materials**).

While GIST is classified as a soft tissue sarcoma, its biology, diagnostic and therapeutic pathways differ in some clinics in Germany substantially from other sarcoma subtypes. In some clinics GISTs are managed by the Department of Gastroenterology or are discussed in tumor boards of the Department of Gastrointestinal Oncology. Nevertheless, according to the German S3 guideline “Adult Soft Tissue Sarcomas”, GIST patients should be managed in, or in consultation with, a sarcoma center and they should be surgically resected by a surgeon with sarcoma and GIST expertise at a specialized sarcoma center (25). The potential bias regarding possible different referral patterns for GIST patients is addressed in an additional comparison between sarcoma patients (excluding GIST) treated within and outside specialized centers. This sensitivity analysis showed that 3,116 patients with bone and soft tissue sarcomas (excluding GIST) were diagnosed in Baden-Württemberg during the study period from 2019 to 2022. Of these, 1,402 (45%) sarcoma patients (excluding GIST) were treated in specialized sarcoma centers, while 1,714 (55%) were treated outside the sarcoma centers.

## 4 Discussion

In our study, we retrospectively analyzed geographic distribution of sarcoma cases incidence in Southwest Germany. Our aim was to depict visually possible underrepresented areas of sarcoma primary diagnoses as white spots by evaluating the data obtained from individual specialized sarcoma centers and comparing them to the number of cases obtained from the Baden-Württemberg Cancer Registry. As a result, we are able to show areas with sarcoma patients treated outside of the five specialized sarcoma centers through depiction of the geographic disparities in sarcoma cases occurrence and treatment in a specialized center. This novel “White-Spot Analysis” of patients diagnosed with sarcoma may help to improve future treatment and treatment outcome for patients. In this context, the term ‘white spot’ does not imply the complete absence of sarcoma cases but rather indicates an underrepresentation of patients treated in specialized centers. We therefore use the term as a descriptive marker of potentially underserved areas.

For the analysis, the postal codes of patients from five specialized sarcoma centers of Baden-Württemberg were used. At the time of data collection (2019–2022), Mannheim and Tübingen were already certified by the German Cancer Society. Due to the large number of sarcoma diagnoses at the other sarcoma centers in Heidelberg, Stuttgart and Freiburg, comparable and adequate sarcoma care can be assumed, even though these centers had not yet been fully reviewed by the German Cancer Society during the study period. However, the sarcoma centers of Heidelberg and Stuttgart have been certified in the meantime by the German Cancer Society, which strengthens our hypothesis.

The “White-Spot Analysis” based on the data from specialized sarcoma centers shows 6 hotspots marked with red rectangles numbered 11–16 showing a high incidence of newly diagnosed sarcoma cases. In contrast, there are 5 broad areas in the east of Baden-Württemberg marked with white rectangles and numbered 1, 2, 4, 6, 9 showing a remarkably low incidence. The major factor could be treatment in border areas by neighboring Federal States such as sarcoma center Ulm, which has not provided individual center data, or in other sarcoma centers outside of Southwest Germany such as the sarcoma center Würzburg, which is located in Bavaria only a few kilometers beyond the north-eastern border to Baden-Württemberg. Thus, we cannot exclude that some patients were treated in neighboring sarcoma centers, which were not captured in our dataset. In addition, individual treatment choices and referral pathways may have influenced where patients received care. This represents an important limitation. Another important aspect to consider is potential treatment of patients with sarcomas in smaller clinics and low-volume centers which are not specialized in sarcoma care. Therefore, we would refrain from further discussing those areas in scope of this paper, as explained above.

The strength of our analysis is that it draws upon sarcoma primary diagnoses from the entire adult population of Baden-Württemberg obtained from the Baden-Württemberg Cancer Registry as well as large patient numbers from 5 individual sarcoma centers. Pediatric sarcoma patients were excluded due to their regular treatment at highly specialized pediatric cancer centers and not necessarily in sarcoma centers in Germany and due to special data protection reasons. Their inclusion would not have reflected adult sarcoma care structures and was therefore beyond the scope of this analysis. Furthermore, our analysis is a novel and innovative approach for studying coverage and medical care of cancer patients. To the best of our knowledge, this is the first and only published “White-Spot Analysis” concerning sarcoma patients and comparing Cancer Registry sarcoma data with individual data from sarcoma centers. Thus, a comparison to established literature is not possible. A “White-Spot Analysis” is a relatively novel method that is used in a wide variety of fields (mostly social and economic sciences) to identify, as the name implies, white spots or gaps in data or information, which can be used to visualize and optimize the corresponding aspect (26). In our real-world study, we aimed to improve sarcoma patient care in Southwest Germany as a result of our “White-Spot Analysis”. We hope to establish White-Spot Analysis as a novel cost-effective tool in epidemiological and public health research for detecting gaps in coverage and optimizing health care in that particular instance.

The data of the study were obtained retrospectively and therefore confined by the typical limitations of a retrospective analysis. In our study, we analyzed the sarcoma patients with primary sarcoma diagnosis who are inhabitants of Baden-Württemberg (data from BWCR) as well as patients that are treated in one of the five sarcoma centers and have place of residence in Baden-Württemberg (individual data from 5 sarcoma centers).

The number of cases and incidence rates were depicted geographically with a map of Baden-Württemberg and a color-coding. In the study we have shown absolute number and incidence of patients with first diagnosis of sarcoma obtained from the German federal Cancer Registry or the sarcoma centers. According to BWCR, we detected districts such as Rottweil, Ostalb and Stuttgart exhibiting the lowest incidence of newly diagnosed sarcoma cases. In contrast, some of the districts with the highest incidence are Bodensee district, Baden-Baden and Rastatt. The Bodensee region exhibited the highest incidence of newly diagnosed sarcoma cases. Some of the factors that might have influenced this phenomenon could be a rather high percentage of population in age groups 45–64 and over 65 years of age with higher incidence of sarcoma but we do not have access to the birth dates of sarcoma patients due to data protection regulations of the BWCR. The district with the second highest number of sarcoma first diagnoses was Baden-Baden. One of the factors that might explain such a high incidence could come through demographical analysis: Baden-Baden was the municipality with the highest percentage of inhabitants over 65 years of age in our study. It is well known that sarcomas have a second peak of incidence in adults above the age of 65 years (2). In addition, another limitation of the analysis may be incomplete reporting of the physicians to the Cancer Registry while the number of unreported cases of sarcoma patients who are neither treated in a sarcoma center nor registered in the Cancer Registry remains unclear. Moreover, the number of patients at postal code levels are in many cases very small so that there could be a distortion of the calculated incidences per 100,000 inhabitants. When interpreting our geographic maps, it is therefore important to note that absolute case numbers in areas with small populations may primarily reflect demographic variation rather than systematic under-referral. For this reason, we reported both absolute counts and incidence rates where feasible. At the postal code level, incidence analysis based on BWCR data was not possible due to data protection regulations and very small case numbers, which must be considered when evaluating potential white spots. Comparing the data of BWCR at district level with the postal codes of the five sarcoma centers it is important to note that some postal code areas belong to more than one district in Germany. However, this affects only 1.7% of the total population of Baden-Württemberg and for data protection reasons the addresses of the sarcoma patients living in these districts and postal code areas are unknown and therefore cannot be subtracted so that this factor must be considered when comparing the incidences.

In our analysis, we could show that 60% of sarcoma patients were treated outside and 40% were managed in specialized sarcoma centers in Baden-Württemberg. Even the sensitivity analysis excluding GIST patients, due to possible differing referral patterns, does not change the fact that more than half of the sarcoma patients (55%) were treated outside, compared to 45% within specialized sarcoma centers in Baden-Württemberg between 2019 and 2022. Thus, although referral patterns for GIST may differ from other sarcomas, our sensitivity analysis confirmed that the overall finding of geographic disparities remains robust, even when GIST are excluded.

In an ideal study, incidence of newly diagnosed sarcoma cases obtained from sarcoma centers should mirror, topographically speaking, the one from the German Federal Cancer Registry. However, when the “White-Spot Analysis” was performed on these data, a different picture can be seen. We observed a series of single or several adjacent white spots throughout western and southwestern Baden-Württemberg. For example, the district of Rastatt exhibited a rather high number of sarcoma patients (11.81 per 100,000 inhabitants per year) according to Baden-Württemberg Cancer Registry. However, a low incidence of sarcoma patients was treated in one of the aforementioned sarcoma centers (3.4 per 100,000 patients per year). In addition, the district of Baden-Baden exhibited a rather high incidence of newly diagnosed sarcoma patients (11.84 per 100,000 inhabitants). This can be seen in the topographic analysis of data obtained from specialized sarcoma centers with mostly low incidence areas (2.63 per 100,000 patients) as well as some white spots with no new cases at all. Another example for such a disparity is the district Bodensee. Here, the highest incidence could be observed (11.9 per 100,000 inhabitants). However, the topographical analysis of those regions based on the data obtained from sarcoma centers shows areas with mostly low incidence of sarcoma patients treated in one of the aforementioned centers (under 5 per 100,000 patients) as well as some white spot areas with no new cases at all. Different factors may play a role. Firstly, a low number of patients may live in a district or postal code area which could potentially result in a random fluctuation of patient numbers between different areas. Secondly, patients treated outside of the five participating sarcoma centers or in a non-specialized low-volume clinic with failure to refer the sarcoma patient to a specialized center, may not have been represented in the data of the “White-Spot Analysis”. In future these data need to be included. Thirdly, the observed discrepancy of sarcoma incidences and treatment at sarcoma centers in regions with an older population could reflect an age disparity, with access to highly specialized medicine being preferably accessible for younger patients. Fourthly, the study period (2019–2022) overlaps with the onset of the COVID-19 pandemic, which may have influenced the diagnosis and treatment of sarcomas as well as the corresponding referral behavior. Nevertheless, oncological care structures in Germany remained functional during the pandemic, and therefore the overall impact on sarcoma management was likely limited, although it cannot be fully excluded.

Our analysis was descriptive and not designed to assess patient outcomes. Survival or treatment efficacy data were not available from the BWCR or from the individual centers. Importantly, it was not our goal to replicate outcome analyses, as superior outcomes of sarcoma management in high-volume centers have already been demonstrated in multiple large international studies, which we have cited extensively. Such analyses were beyond the scope of our study. Accordingly, our conclusions focus on geographic disparities rather than outcome differences.

As previously discussed, it is strongly recommended that a specialized sarcoma center should be involved in the diagnostic and treatment algorithm of patients diagnosed with sarcoma, due to the rarity and complexity of the disease (9, 10, 14). Multiple large studies yielded some quality evidence of superior outcomes for tumor patients that receive an interdisciplinary treatment in the scope of specialized high-volume and possibly certified tumor centers for example according to German Cancer Society (DKG) which is not only important for sarcomas (8, 9, 14) but also for other tumors like lung, pancreas or colon cancer (27–31). Some of the potential advantages of treatment in specialized centers are for example a more experienced team involved in the treatment of sarcoma, interdisciplinary tumor boards to decide further diagnostic measures, neoadjuvant/local treatment possibilities (for example loco-regional deep hyperthermia, radiotherapy or isolated limb perfusion), availability of advanced diagnostic methods (such as MRI, PET-CT or tumor genome sequencing with molecular tumor boards in centers of personalized medicine), potential participation in clinical studies and as a consequence overall more treatment options for every sarcoma patient (9, 14). Moreover, incorrect sarcoma diagnoses were found in up to 40% of patient cases in several studies, thereby indicating that a centralized or expert pathology review could significantly improve both diagnostic accuracy and subsequent treatment quality (13). Beyond geographic factors, systemic aspects of the German healthcare structure may also contribute to the observed disparities. These include the role of certified Comprehensive Cancer Centers (CCCs), established referral pathways for rare cancers, and potential reimbursement-related or structural barriers. Such system-level determinants may partly explain why some patients are not referred to sarcoma centers, even when such centers are geographically accessible.

To address the disparities and to increase the percentage of treatment in specialized sarcoma centers, we would like to highlight further distribution of relevant sarcoma information for example through homepages of sarcoma expert centers, mailing lists, leaflets, training seminars and referring physician events for oncologists, general practitioners and surgeons in both clinics and outpatient departments or doctors’ offices. Moreover, patients and doctors should take part in sarcoma support groups and sarcoma centers in order to raise awareness among both physicians and patients for the rare entity of sarcomas and the importance for treatment in specialized sarcoma centers in terms of better patient outcome. In addition, sarcoma centers should collaborate more with private foundations such as the German Sarcoma Foundation due to their large patient outreach.

To summarize, novel “White-Spot Analysis” is a useful tool for visualization of disparities in sarcoma care. While it does not establish causality, it can help optimize the access of sarcoma patients to specialized high-volume centers and to minimize the observed discrepancies between the treatment in specialized high-volume sarcoma centers and non-specialized care in Southwest Germany. In the future, the main goal is to change sarcoma medical care systemically in order to improve the limited prognosis and outcome for each patient with such a rare and complex disease. Future studies extending this approach to larger regions may further clarify national patterns and support systemic improvements in sarcoma care.

## 5 Conclusion

This multicenter analysis combining cancer registry data and in-house patient numbers from five sarcoma centers demonstrates substantial geographic disparities in access to specialized sarcoma care in Baden-Württemberg. Only 40% of patients were treated in expert centers, with clear regional variation and underrepresented areas identified by White-Spot Analysis. These findings emphasize the urgent need to strengthen referral pathways, enhance collaboration across centers and regions, and raise awareness among both physicians and patients. White-Spot Analysis offers a cost-effective and reproducible tool to visualize inequities and to guide future strategies for improving equitable access to high-quality sarcoma care. This analysis did not assess survival or treatment efficacy. Future work should link geography to outcome endpoints to quantify clinical impact.

## Data Availability

The original contributions presented in the study are included in the article/**Supplementary Material**. Further inquiries can be directed to the corresponding author.

## Supplementary material

Supplementary Table 1 Sarcoma histologies with ICD-O codes according to the WHO classification (2019–2022, Baden-Württemberg Cancer Registry). The table lists all histological subtypes included in the study, their ICD codes, absolute frequencies, and percentages among the study population.

Supplementary Table 2 Number of newly diagnosed sarcoma patients according to district codes of the German Federal State of Baden-Württemberg, 2019–2022, based on data from the Baden-Württemberg Cancer Registry (BWCR). Columns include district code, population size, area, absolute case numbers, crude incidence per 100,000 inhabitants per year, numbers treated in one of the five specialized sarcoma centers, and numbers treated outside specialized centers (calculated as BWCR minus center data).

Supplementary Table 3 Number of newly diagnosed sarcoma patients with residence in Baden-Württemberg treated in one of the five specialized sarcoma centers, 2019–2022. Columns include absolute patient numbers per center, maximum patient numbers per postal code area, average and median patient numbers per postal code population, and rates per 100,000 inhabitants (with interquartile ranges where applicable).
