# Correction: An analysis of the distribution of bone and soft tissue sarcoma diagnoses and their disparities in Southwest Germany: a multicenter approach

**DOI:** 10.3389/fonc.2025.1730234

**Published:** 2025-11-06

**Authors:** Branko Calukovic, Katrin Benzler, Mary E. Carter, Adrien Daigeler, Johannes T. Thiel, Jens Jakob, Bernd Kasper, Gerlinde Egerer, Leonidas Apostolidis, David Braig, Simone Hettmer, Claudia Blattmann, Markus Knott, Lars Zender, Christoph K. W. Deinzer

**Affiliations:** 1 Department of Internal Medicine VIII - Medical Oncology and Pneumology, Medical University Hospital Tübingen, Tübingen, Germany; 2 Center for Soft Tissue Sarcomas, GIST and Bone Tumors (ZWS) of the University Hospital Tübingen and the Comprehensive Cancer Center (CCC) Tübingen-Stuttgart, Tübingen, Germany; 3 DFG Cluster of Excellence 2180 ‘Image-Guided and Functional Instructed Tumor Therapy’ (iFIT), University of Tübingen, Tübingen, Germany; 4 Department of Hand, Plastic, Reconstructive and Burn Surgery, BG-Unfallklinik Tübingen, Eberhard Karls University of Tübingen, Tübingen, Germany; 5 Sarcoma Unit, Mannheim University Medical Center, University of Heidelberg, Mannheim, Germany; 6 Sarcoma Center Heidelberg, Heidelberg, Germany; 7 Department of Hematology, Oncology and Rheumatology, University Hospital Heidelberg, Heidelberg, Germany; 8 Department of Medical Oncology, National Center for Tumor Diseases (NCT) Heidelberg, Heidelberg University Hospital, Heidelberg, Germany; 9 Sarcoma Center of the Comprehensive Cancer Center Freiburg (CCCF), Freiburg, Germany; 10 Department of Plastic and Hand Surgery, Medical Center - University of Freiburg, Faculty of Medicine, University of Freiburg, Freiburg, Germany; 11 Division of Pediatric Hematology and Oncology, Department of Pediatric and Adolescent Medicine, University Medical Center Freiburg, University of Freiburg, Freiburg, Germany; 12 Sarcoma Center Stuttgart Cancer Center (SCC), Klinikum Stuttgart, Stuttgart, Germany; 13 Department of Paediatric Hematology and Oncology, Olgahospital, Stuttgart Cancer Center (SCC), Klinikum Stuttgart, Stuttgart, Germany; 14 Stuttgart Cancer Center - Tumorzentrum Eva Mayr-Stihl, Klinikum Stuttgart, Stuttgart, Germany; 15 Department of Hematology and Oncology, Klinikum Stuttgart, Stuttgart, Germany

**Keywords:** soft tissue sarcoma, bone sarcoma, white-spot analysis, cancer registry, geographic disparities, specialized sarcoma center, real world data

There was a mistake in **Figure 3** as published. By mistake, all five maps in **Figure 3** showed only the sarcoma primary cases of the Sarcoma Center Stuttgart. The corrected **Figure 3** appears below.

**Figure 3:**
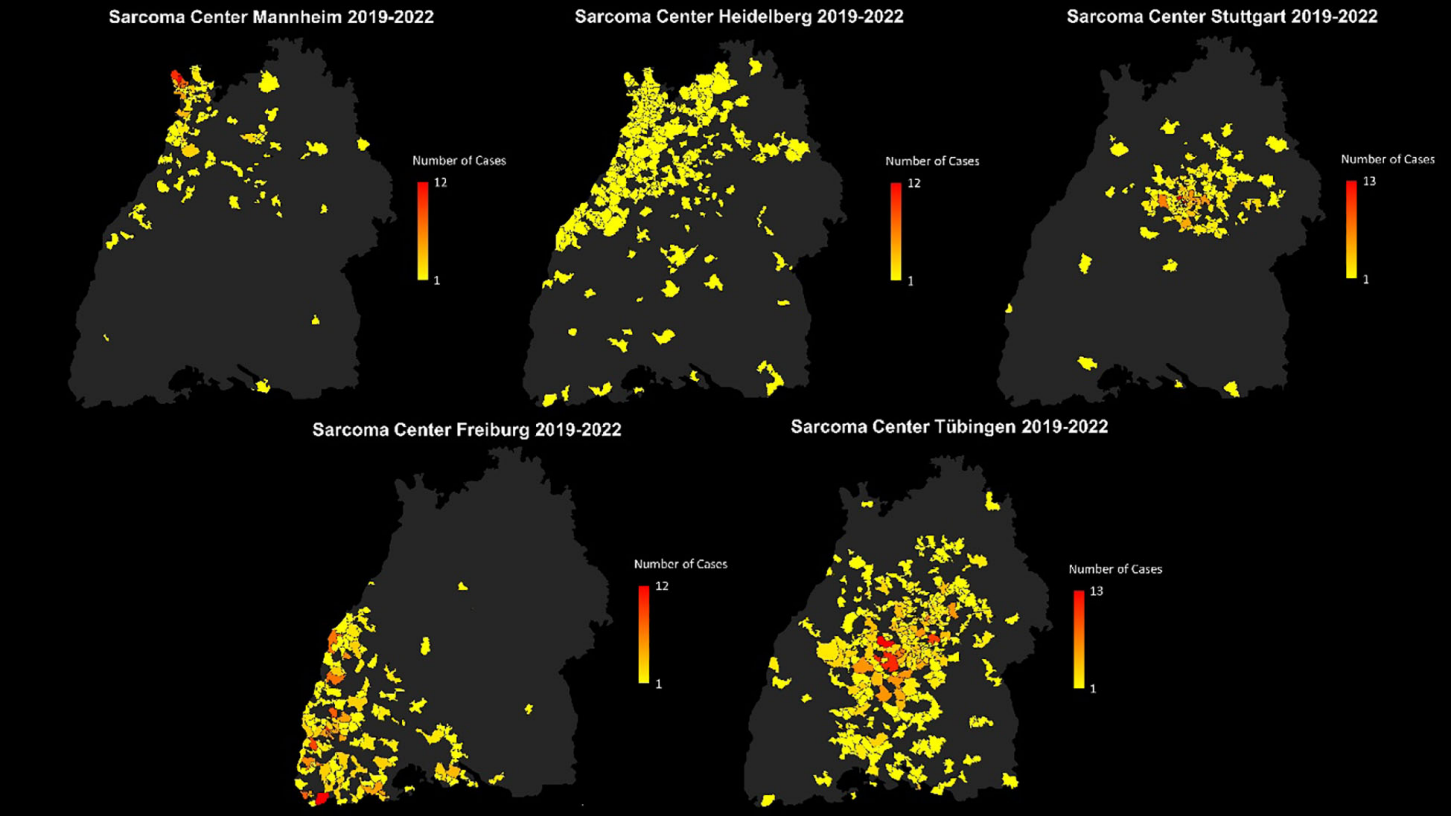
Distribution of patients with sarcoma first diagnoses treated in the sarcoma centers Mannheim, Heidelberg, Stuttgart, Freiburg and Tübingen with absolute patient numbers across the postal code areas. Each center is displayed individually to visualize its specific catchment area.

The original version of this article has been updated.

