# Supplementary material for: An analysis of the distribution of bone and soft tissue sarcoma diagnoses and their disparities in Southwest Germany: a multicenter approach
Source: Front Oncol. 2025 Oct 22;15:1592004. doi: 10.3389/fonc.2025.1592004 (PMC12586119; doi:10.3389/fonc.2025.1592004)
Supplement: Supplementary Table 1 — Sarcoma histologies with ICD-O codes according to the WHO classification (2019–2022, Baden-Württemberg Cancer Registry). The table lists all histological subtypes included in the study, their ICD codes, absolute frequencies, and percentages among the study population. [file Table1.docx]

## Supplementary Materials

## Supplementary Table 1. Sarcoma histologies with ICD-O codes according to the WHO classification (2019–2022, Baden-Württemberg Cancer Registry). The table lists all histological subtypes included in the study, their ICD codes, absolute frequencies, and percentages among the study population.

| **Histology Code** | **Histology** | **Frequency** | **Percentage** |
| --- | --- | --- | --- |
| 8714/3 | Uterine PEComa | 2 | 0,05% |
| 8800/3 | Sarcoma NOS | 122 | 2,99% |
| 8801/3 | Spindle cell sarcoma | 54 | 1,32% |
| 8802/3 | Giant cell sarcoma | 505 | 12,36% |
| 8803/3 | Small cell sarcoma | 4 | 0,1% |
| 8804/3 | Epithelioid sarcoma | 17 | 0,42% |
| 8805/3 | Undifferentiated uterine sarcoma | 110 | 2,69% |
| 8806/3 | Desmoplastic small round cell tumor (DSRCT) | 4 | 0,1% |
| 8810/3 | Fibrosarcoma | 29 | 0,71% |
| 8811/3 | Myxofibrosarcoma | 125 | 3,06% |
| 8813/3 | Fibrosarcoma of the fascia | 1 | 0,02% |
| 8815/3 | Solitary fibrous tumor | 36 | 0,88% |
| 8832/3 | Dermatofibrosarcoma protuberans | 115 | 2,81% |
| 8840/3 | Myxosarcoma | 8 | 0,2% |
| 8850/3 | Liposarcoma NOS | 106 | 2,59% |
| 8851/3 | Well-differentiated liposarcoma | 93 | 2,28% |
| 8852/3 | Myxoid liposarcoma | 81 | 1,98% |
| 8853/3 | Round cell liposarcoma | 2 | 0,05% |
| 8854/3 | Pleomorphic liposarcoma | 30 | 0,73% |
| 8855/3 | Mixed cell liposarcoma | 1 | 0,02% |
| 8858/3 | Dedifferentiated liposarcoma | 155 | 3,79% |
| 8890/3 | Leiomyosarcoma NOS | 353 | 8,64% |
| 8891/3 | Epithelioid leiomyosarcoma | 13 | 0,32% |
| 8896/3 | Myxoid leiomyosarcoma | 1 | 0,02% |
| 8900/3 | Rhabdomyosarcoma NOS | 20 | 0,49% |
| 8901/3 | Adult pleomorphic rhabdomyosarcoma | 18 | 0,44% |
| 8910/3 | Embryonal rhabdomyosarcoma NOS | 2 | 0,05% |
| 8912/3 | Spindle cell rhabdomyosarcoma | 3 | 0,07% |
| 8920/3 | Alveolar rhabdomyosarcoma | 8 | 0,2% |
| 8930/3 | Endometrial stromal sarcoma | 59 | 1,44% |
| 8931/3 | Low-grade endometrial stromal sarcoma | 33 | 0,81% |
| 8936/3 | Gastrointestinal stromal tumor (GIST) | 971 | 23,76% |
| 8963/3 | Extrarenal rhabdoid tumor | 3 | 0,07% |
| 8964/3 | Clear cell sarcoma of the kidney | 1 | 0,02% |
| 8980/3 | Carcinosarcoma NOS | 303 | 7,41% |
| 9040/3 | Synovial sarcoma NOS | 38 | 0,93% |
| 9041/3 | Spindle cell synovial sarcoma | 15 | 0,37% |
| 9043/3 | Biphasic synovial sarcoma | 11 | 0,27% |
| 9044/3 | Clear cell sarcoma NOS | 12 | 0,29% |
| 9045/3 | Biphenotypic sinonasal Sarcoma | 1 | 0,02% |
| 9120/3 | Angiosarcoma | 168 | 4,11% |
| 9133/3 | Epithelioid hemangioendothelioma | 15 | 0,37% |
| 9137/3 | Intimal sarcoma | 10 | 0,24% |
| 9140/3 | Kaposi sarcoma | 95 | 2,32% |
| 9170/3 | Lymphangiosarcoma | 1 | 0,02% |
| 9180/3 | Osteosarcoma NOS | 64 | 1,57% |
| 9181/3 | Chondroblastic osteosarcoma | 5 | 0,12% |
| 9184/3 | Osteosarcoma in Paget´s Disease of bone | 1 | 0,02% |
| 9187/3 | Low-grade central osteosarcoma | 1 | 0,02% |
| 9192/3 | Parosteal osteosarcoma | 3 | 0,07% |
| 9193/3 | Periosteal osteosarcoma | 1 | 0,02% |
| 9194/3 | High-grade surface osteosarcoma | 1 | 0,02% |
| 9220/3 | Chondrosarcoma NOS | 83 | 2,03% |
| 9221/3 | Periosteal chondrosarcoma | 2 | 0,05% |
| 9231/3 | Myxoid chondrosarcoma | 14 | 0,34% |
| 9240/3 | Mesenchymal chondrosarcoma | 9 | 0,22% |
| 9242/3 | Clear cell chondrosarcoma | 1 | 0,02% |
| 9243/3 | Dedifferentiated chondrosarcoma | 4 | 0,1% |
| 9250/3 | Giant cell tumor of the bone | 1 | 0,02% |
| 9252/3 | Tenosynovial giant cell tumor | 4 | 0,1% |
| 9364/3 | Ewing sarcoma | 45 | 1,1% |
| 9370/3 | Chordoma | 48 | 1,17% |
| 9540/3 | Malignant peripheral nerve sheath tumor (MPNST) | 40 | 0,98% |
| 9542/3 | Epithelioid peripheral nerve sheath tumor | 2 | 0,05% |
| 9581/3 | Alveolar soft tissue sarcoma | 2 | 0,05% |
| 9561/3 | Triton tumor | 2 | 0,05% |
| **Sum** |  | **4087** | **100%** |

## Supplementary Table 2. Number of newly diagnosed sarcoma patients according to district codes of the German Federal State of Baden-Württemberg, 2019–2022, based on data from the Baden-Württemberg Cancer Registry (BWCR). Columns include district code, population size, area, absolute case numbers, crude incidence per 100,000 inhabitants per year, numbers treated in one of the five specialized sarcoma centers, and numbers treated outside specialized centers (calculated as BWCR minus center data).

| **Municipality/ District** | **District Code** | **Number of inhabitants** | **Area in km^2^** | **Absolute number of new sarcoma diagnoses according to BWCR 2019-2022** | **Incidence per 100,000 inhabitants per year according to BWCR data** | **Absolute number of sarcoma diagnoses treated in one of 5 specialized sarcoma centers 2019-2022** | **Patients treated outside of 5 specialized sarcoma centers (Difference of BWCR and sarcoma center data)** |
| --- | --- | --- | --- | --- | --- | --- | --- |
| **Stuttgart** | 08111 | 632865 | 207.33 | 163 | 6.44 | 115 | 48 |
| **Böblingen** | 08115 | 398528 | 617.77 | 143 | 9.00 | 75 | 68 |
| **Esslingen** | 08116 | 540226 | 641.28 | 226 | 10.46 | 108 | 118 |
| **Göppingen** | 08117 | 261857 | 642.34 | 107 | 10.22 | 17 | 90 |
| **Ludwigsburg** | 08118 | 551051 | 686.77 | 188 | 8.53 | 70 | 118 |
| **Rems-Murr** | 08119 | 432397 | 858.08 | 173 | 10.00 | 91 | 82 |
| **Heilbronn City** | 08121 | 128334 | 99.89 | 50 | 9.74 | 11 | 39 |
| **Heilbronn District** | 08125 | 353283 | 1099.91 | 112 | 7.93 | 41 | 71 |
| **Hohenlohe** | 08126 | 115063 | 776.75 | 43 | 9.34 | 7 | 36 |
| **Schwäbisch Hall** | 08127 | 202834 | 1484.08 | 63 | 7.76 | 12 | 51 |
| **Main-Tauber** | 08128 | 134745 | 1304.12 | 40 | 7.42 | 3 | 37 |
| **Heidenheim** | 08135 | 135035 | 627.14 | 48 | 8.89 | 1 | 47 |
| **Ostalb** | 08136 | 319631 | 1511.39 | 82 | 6.41 | 15 | 67 |
| **Baden-Baden** | 08211 | 57025 | 140.19 | 27 | 11.84 | 6 | 21 |
| **Karlsruhe City** | 08212 | 308707 | 173.42 | 118 | 9.56 | 32 | 86 |
| **Karlsruhe District** | 08215 | 454613 | 1084.98 | 189 | 10.39 | 67 | 122 |
| **Rastatt** | 08216 | 234981 | 738.43 | 111 | 11.81 | 32 | 79 |
| **Heidelberg** | 08221 | 162273 | 108.83 | 55 | 8.47 | 52 | 3 |
| **Mannheim** | 08222 | 315554 | 144.97 | 111 | 8.79 | 83 | 28 |
| **Neckar-Odenwald** | 08225 | 145493 | 1125.95 | 45 | 7.73 | 23 | 22 |
| **Rhein-Neckar** | 08226 | 555352 | 1061.55 | 176 | 7.92 | 136 | 40 |
| **Pforzheim** | 08231 | 127849 | 97.99 | 55 | 10.75 | 15 | 40 |
| **Calw** | 08235 | 162853 | 797.29 | 63 | 9.67 | 23 | 40 |
| **Enz** | 08236 | 202536 | 573.6 | 72 | 8.89 | 28 | 44 |
| **Freudenstadt** | 08237 | 121164 | 870.4 | 48 | 9.90 | 28 | 20 |
| **Freiburg** | 08311 | 236140 | 153.04 | 101 | 10.69 | 52 | 49 |
| **Breisgau-Hochschwarzwald** | 08315 | 269948 | 1378.32 | 78 | 7.22 | 53 | 25 |
| **Emmendingen** | 08316 | 170996 | 679.8 | 58 | 8.48 | 29 | 29 |
| **Ortenau** | 08317 | 441885 | 1860.29 | 167 | 9.45 | 63 | 104 |
| **Rottweil** | 08325 | 142593 | 769.42 | 34 | 5.96 | 18 | 16 |
| **Schwarzwald-Baar** | 08326 | 217181 | 1025.34 | 67 | 7.71 | 24 | 43 |
| **Tuttlingen** | 08327 | 144891 | 734.38 | 52 | 8.97 | 19 | 33 |
| **Konstanz** | 08335 | 292568 | 817.98 | 132 | 11.28 | 33 | 99 |
| **Lörrach** | 08336 | 233027 | 806.66 | 76 | 8.15 | 45 | 31 |
| **Waldshut** | 08337 | 173460 | 1131.15 | 66 | 9.51 | 38 | 28 |
| **Reutlingen** | 08415 | 291696 | 1092.48 | 100 | 8.57 | 54 | 46 |
| **Tübingen** | 08416 | 232803 | 519.12 | 91 | 9.77 | 61 | 30 |
| **Zollernalb** | 08417 | 193235 | 917.58 | 68 | 8.80 | 35 | 33 |
| **Ulm** | 08421 | 128928 | 118.68 | 56 | 10.86 | 2 | 54 |
| **Alb-Donau** | 08425 | 202476 | 1358.55 | 83 | 10.25 | 2 | 81 |
| **Biberach** | 08426 | 206513 | 1409.52 | 77 | 9.32 | 5 | 72 |
| **Bodensee** | 08435 | 222712 | 664.77 | 106 | 11.90 | 8 | 98 |
| **Ravensburg** | 08436 | 290911 | 1632.08 | 119 | 10.23 | 8 | 111 |
| **Sigmaringen** | 08437 | 134045 | 1204.23 | 48 | 8.95 | 10 | 38 |
|  |  | **Sum 11,280,257** | **Sum 35,747.85** | **Sum 4087** | **Average 9.18** | **Sum 1650** | **Sum 2437** |

## Supplementary Table 3. Number of newly diagnosed sarcoma patients with residence in Baden-Württemberg treated in one of the five specialized sarcoma centers, 2019–2022. Columns include absolute patient numbers per center, maximum patient numbers per postal code area, average and median patient numbers per postal code population, and rates per 100,000 inhabitants (with interquartile ranges where applicable).

| **Sarcoma Primary Cases with residence in Baden-Württemberg treated between 2019-2022 at Sarcoma Center** | **Absolute Patient Numbers (n)** | **Maximum Patient Numbers per Postal Code Area** | **Average Patient Numbers per Postal Code Population (n)** | **Average Patient Numbers per Postal Code Population (n/100,000)** | **Median Patient Numbers per Postal Code Population (n) [IQR]** | **Median Patient Numbers per Postal Code Population (n/100,000)**  **[IQR]** |
| --- | --- | --- | --- | --- | --- | --- |
| **Freiburg** | 301 | 12 | 2.3 | 30.5 | 1 [2] | 24.0 [21.2] |
| **Heidelberg** | 415 | 12 | 2.0 | 18.1 | 1 [1] | 14.8 [14.2] |
| **Mannheim** | 154 | 12 | 1.9 | 12.6 | 1 [0] | 9.7 [10.0] |
| **Stuttgart** | 253 | 13 | 2.1 | 16.1 | 1 [1] | 12.8 [12.9] |
| **Tübingen** | 527 | 13 | 1.9 | 22.0 | 1 [1] | 15.7 [17.8] |
| **Sum of Cases** | **1650** |  |  |  |  |  |
